# Flow and ischemic changes in retina and choroid across diabetic retinopathy spectrum: a SS-OCTA study

**DOI:** 10.1038/s41433-025-03639-y

**Published:** 2025-02-27

**Authors:** Qianhui Yang, Kelvin Y. C. Teo, Yueheng Hong, Bingyao Tan, Leopold Schmetterer, Chui Ming Gemmy Cheung, Tien Yin Wong, Gavin Tan Siew Wei

**Affiliations:** 1 Tianjin Key Laboratory of Retinal Functions and Diseases, Tianjin Branch of National Clinical Research Center for Ocular Disease, Eye Institute and School of Optometry, Tianjin Medical University Eye Hospital, Tianjin, China; 2 Singapore National Eye Centre, Singapore Eye Research Institute, Singapore, Republic of Singapore; Duke-NUS Medical School, Singapore, Republic of Singapore; 3 Ophthalmology & Visual Sciences Academic Clinical Program, Duke-NUS Medical School, Singapore, Republic of Singapore; 4 SERI-NTU Advanced Ocular Engineering (STANCE), Singapore, Republic of Singapore; 5 School of Chemical and Biological Engineering, Nanyang Technological University, Singapore, Republic of Singapore; 6 Center for Medical Physics and Biomedical Engineering, Medical University Vienna, Vienna, Austria; 7 Department of Clinical Pharmacology, Medical University Vienna, Vienna, Austria; 8 Institute of Molecular and Clinical Ophthalmology, Basel, Switzerland; 9 Foundation Ophtalmologique Adolphe De Rothschild, Paris, France; 10 Tsinghua Medicine, Tsinghua University, Beijing, China

**Keywords:** Retinal diseases, Diabetes complications

## Abstract

**Purpose:**

To examine changes in retinal and choroidal vasculature in diabetes mellitus across the range of diabetic retinopathy (DR) severities using optical coherence tomography angiography (OCTA) and compare the patterns of vascular changes.

**Methods:**

We conducted a cross-sectional study enrolling 296 patients (498 eyes) with diabetes mellitus. Swept-Source OCT Angiography variables in both retina and choroid, including perfusion density (PD), vessel density (VD), large vessel density (LVD) in both superficial and deep layer of retina and CC flow voids (FD) density of the choroid were quantified. Correlations between OCTA parameters and DR severity, visual acuity and studied factors were performed.

**Results:**

Totally 498 eyes including 176 had no DR, 160 had mild NPDR, 98 had moderate NPDR, 11 had severe NPDR, 41 had PDR with PRP, and 12 had PDR without PRP. Choriocapillaris (CC) flow voids density increased with increasing DR severity (17.06% vs 17.41% vs 17.60% vs 17.62% vs 18.05% vs 18.41%, p-trend = 0.0004), FAZ area increased with DR severity in both superficial and deep layer (superficial layer p trend=0.0027; deep layer p trend=0.0022). Visual acuity correlated negatively with CC flow voids (Pearson’s ρ = 0.09, p = 0.04) and superficial FAZ area (Pearson’s ρ = 0.22, *p* < 0.001), while inversely correlated with SCP PD (Pearson’s ρ = −0.15, *p* < 0.001) and VD (Pearson’s ρ = −0.15, *p* < 0.001), as well as DCP PD (Pearson’s ρ = −0.21, *p* < 0.001) and VD (Pearson’s ρ = −0.19, *p* < 0.001).

**Conclusion:**

Choriocapillaris ischemia increased, FAZ area enlarged, and total retina perfusion density decreased with increasing DR severity. The deep layer and large vessels may change in early stage before DR progresses to PDR. More ischemia and vessel tortuosity are correlated with worse visual acuity and higher HbA1c level. OCTA can be utilized to detect both large and small vascular changes in both the retina and choroid in DR patients.

## Introduction

As the most common microvascular complication of diabetes mellitus (DM), diabetic retinopathy (DR) has evolved into a global-health problem which major visual impact on the working age population [1–3]. It has been estimated the global population with DM will rise to 700 million in 2045, 30–40% of them will have DR [4, 5]. Current DR classification methods are based on traditional color fundus photographs by modified Airlie House/Early Treatment Diabetic Retinopathy Study (ETDRS) or International Classification [6, 7]. However, subtle microvascular changes such as small non perfusion area and vessel distortion in the retina and choroid across different layers cannot be detected by color fundus photographs alone [8, 9]. Early DR patients with subtle changes may require closer monitoring or referral to retina specialists to prevent DR progression. However, some minor early changes are often overlooked. Furthermore, the changes in retinal and choroidal vessels for DR progression remain unclear. OCTA can help bridge this gap by detecting early changes and providing further insights into the progression of vascular alterations across different stages of diabetic retinopathy severity [10, 11].

OCTA is a non-invasive imaging method used to detect microvascular abnormalities across the retina and choroid in DR patients [12, 13] It can be used to measure parameters, like foveal avascular zone (FAZ) area, FAZ circularity, and perfusion density, numerous studies have showed its utility in assessing the severity of DR and increasing the understanding of retinal microvascular disruption in diabetes [14–16]. Previous studies using OCTA have demonstrated that larger FAZ area correlates with DR progression [17] as well as decrease in central visual function [18, 19]. FAZ circularity was also found to be more sensitive than the FAZ area for DR severity due to lower inter-person variability [20]. Using OCTA to measure vessel perfusion area in the central retina, several studies showed that decreasing perfusion density correlated with both NPDR and PDR severity [21, 22].Swept-source OCTA (SS-OCTA) can provide superior visualization of the vasculature beneath Bruch’s membrane, enabling quantification of choriocapillaris not seen in FA and ICGA [23]. Current studies have identified correlations between choroidal vascular changes and DR severity [12, 24]. Extracting quantitative choriocapillaris (CC) features with an automated algorithm, our group previously demonstrated that CC parameters could better discriminate severity of DR in DM patients, compared to the using retinal vessel parameters alone [25]. Other studies have reported in CC flow defects in the FAZ of DM patients DM, with presence of DR [21, 26, 27], however few have correlated these CC features with DR severity.

The purpose of our study is to investigate quantitative features in both large and small vessels in the retinal and choroidal layers using OCTA in patients at all stages of DR. Additionally, we aim to assess the correlation between these vascular parameters and patients’ systemic parameters.

## Methods

Our study was a prospectively recruited clinical study of patients with diabetes mellitus from a single tertiary eye center. Patients with different DR levels, were enrolled from Singapore National Eye Center (SNEC) clinics from April 2018 to July 2019. All the participants signed the informed consent forms based on requirements of Declaration of Helsinki, and the study approval from Singapore Eye Research Institute’s institutional review board. This study followed the Strengthening the Reporting of Observational Studies in Epidemiology (STROBE) reporting guideline.

For this eye-level analysis, each eye was classified according to the International Clinical Disease Severity Scale for DR into 6 groups: no DR, mild NPDR, moderate NPDR, severe NPDR, PDR with PRP and PDR without PRP [7]. In this study, the “no DR” group included diabetic patients who showed no clinical signs of DR on examination. For those who underwent PRP, we presumed that they had previously experienced PDR. The inclusion criteria for DM patients were: (1) age ≥21 years old, (2) type 2 diabetes >5 years (3) No end stage of renal failure. Exclusion criteria were glaucoma, age-related macular degeneration, significant media opacity. IOL Master 700 measured the axial eye length, and eyes longer than 26.5 mm were also excluded. Imaging parameters of vascular changes in the retina and choroid on OCTA (outlined below) were subsequently compared between groups.

### Optical coherence tomography angiography

We utilized a prototype SS-OCTA system (PlexElite 9000, Zeiss Meditec, Dublin, California, USA) with a wavelength scanning laser (λc = 1050 nm) as the light source. The system operates at a speed of 200kHz A-scan/s, with axial and lateral resolutions of 6.3 μm and 20 μm in tissue, respectively.

All scans were conducted by experienced ophthalmic technicians at SNEC. The specific details of OCTA procedures have been described previously [13]. A 3 × 3 mm^2^ area was scanned centered at the fovea. OCTA images were generated using a microangiography algorithm [28]. We utilized PlexElite Review Software (Version 1.6, Zeiss Meditec) automatically segmented for the retina layers and retinal pigment epithelium (RPE). Incorrect auto-segmentations were corrected manually. And CC layers segmentations were performed by a standard protocol developed by Spaide (31–39 μm underneath RPE). Images with poor quality, weak signals (signal strength less than 7) and ungradable artefacts will be excluded followed by standard criteria [29].

### OCTA image analysis

We performed the quantitative analysis for OCTA images by using a custom MATLAB algorithm (The MathWorks Inc). A combined Gabor and Hessian-based vessel filter was utilized to enhance the contrast of large vessels, then a thresholding method was used to generate a binarization mask of large vessels. Both superficial and deep vascular plexus foveal avascular zone (FAZ) area were manually delineated, FAZ size and perimeter were extracted. Next, we calculated following 4 vascular perimeters: perfusion density (PD), vessel density (VD), large vessel density (LVD), CC flow voids (FD) density. PD in the annulus was assessed as the perfused area divided by the total annulus area. VD in each annulus was determined by the total vessel length per total annulus area. CC FD refers to areas of absence of detectable blood flow in choriocapillaris layer, and we calculated the CC FD for various size thresholds ( > 200 μm², >400 μm², >600 μm², and >800 μm²) separately. Using the binarization mask, we calculated PD and VD with and without large vessels. The CC FD density was determined by calculating the percentage of the image area occupied by flow voids, excluding regions with large vessels. For PD, VD, and Large Vessel Density (LVD), higher values indicated better vessel integrity, while higher FD values indicated worse vascular distribution. We extracted CC vessel information by using its morphological images. Binarization was performed by a threshold of mean-SD [30]. The detailed protocol has been published in another paper by our team [12].

### Statistical analysis

We analysed the data by using R (Version 4.4.0). Participants’ baseline characteristics were reported as means ± standard deviations (SD) for continuous variables and numbers and percentages (%) for categorical variables. Retinal and choroidal vascular metrics were analysed between six DR severity groups (no DR, mild NPDR, moderate NPDR, severe NPDR, PDR with PRP, PDR without PRP). Shapiro-Wilk test was used to assess normality of the metrics. We assessed the trend across DR categories of OCTA variable by calculating the p-trend value. Additionally, simple linear regression was employed to analyse differences between individual groups. All means used in the p-trend test were all adjusted by age. Adjusted OCTA means were reported as means (standard error). Comparisons between groups were conducted using ANOVA or Kruskal-Wallis test for continuous variables, and Chi-Square test or Fisher’s exact test for categorical variables.

We examined partial correlations (adjusted for Age and DR severity) between visual acuity, logMAR, HbA1C, DM duration, axial length, spherical equivalent (D), and OCTA parameters. Sub-group analysis for OCTA metrics was conducted within the severe NPDR, PDR with PRP, PDR without PRP groups. Pairwise comparisons were done on severe NPDR, PDR with PRP, PDR without PRP groups using *t* test or Wilcoxon rank sum test depending on the normality of the data. *P* values less than 0.05 were considered statistically significant.

## Results

A total of 498 eyes from 296 type 2 DM patients with different DR levels, were enrolled from Singapore National Eye Center clinic in our study. Table 1 shows the baseline characteristics between groups. We observed significant differences in age, race, duration of diabetes mellitus (DM), HbA1C levels, serum glucose levels, estimated glomerular filtration rate (eGFR), logMAR visual acuity (VA) and axial length between 6 groups. In our dataset, age varied significantly between groups (65.31 ± 8.34 vs 62.58 ± 9.46 vs 60.67 ± 9.93 vs 52.18 ± 6.48 vs 63.66 ± 9.96 vs 54 ± 8.41years, in the no, mild, moderate, severe, PDR with PRP and PDR without PRP DR groups respectively, *p* < 0.0001), patients in severe NPDR group were youngest among groups. Meanwhile, DM duration varied between groups (16.52 vs 19.56 vs 16.02 vs 14.0 vs 23.27 vs 17.58 years; respectively; *p* = 0.0001), among which PDR with PRP group had the longest duration. When comparing the variations of HbA1C, glucose serum and eGFR between groups severe NPDR group exhibited the highest level (Table 1).

**Table 1:** Characteristics and DR severity of patients.

Demographics	No DR (*n* = 176)	Mild NPDR (*n* = 160)	Moderate NPDR (*n* = 98)	Severe NPDR (*n* = 11)	PDR with PRP (*n* = 41)	PDR without PRP (*n* = 12)	Total (*n* = 498)	*p* value
Age (years, mean ± SD)	65.31 ± 8.34	62.58 ± 9.46	60.67 ± 9.93	52.18 ± 6.48	63.66 ± 9.96	54.00 ± 8.41	65.31 ± 8.34	**<0.001**
Gender (male %)	117 (66.5)	110 (68.8)	70 (71.4)	7 (63.6)	28 (68.3)	10 (83.3)	342 (68.7)	0.857
Race (%)								
Chinese	154 (87.5)	133 (83.1)	69 (70.4)	7 (63.6)	32 (78.0)	6 (50.0)	154 (87.5)	**<0.001**
Indian	13 (7.4)	18 (11.2)	12 (12.2)		6 (14.6)	5 (41.7)	13 (7.4)	
Malay	8 (4.5)	8 (5.0)	15 (15.3)	4 (36.4)	3 (7.3)	1 (8.3)	8 (4.5)	
Others	1 (0.6)	1 (0.6)	2 (2.0)				1 (0.6)	
Duration of DM (years)	16.52 (10.26)	19.56 (9.99)	16.02 (8.36)	14.00 (7.55)	23.27 (9.52)	17.58 (9.66)	16.52 (10.26)	**<0.001**
glucose serum (mmol/L)	9.44 (4.12)	10.14 (5.13)	11.22 (4.20)	13.02 (5.61)	10.24 (3.92)	12.40 (3.72)	10.25 (4.53)	**0.0023**
HbA1C (%)	7.37 (0.97)	8.10 (1.47)	8.77 (1.91)	9.52 (1.77)	8.00 (1.23)	9.07 (1.16)	8.02 (1.51)	**<0.001**
MAP (mmHg)	97.38 (12.07)	97.41 (12.73)	96.10 (12.35)	106.50 (19.79)	96.91 (14.61)	103.65 (18.94)	97.45 (13.00)	0.186
glucose serum (mmol/L)	9.44 (4.12)	10.14 (5.13)	11.22 (4.20)	13.02 (5.61)	10.24 (3.92)	12.40 (3.72)	10.25 (4.53)	**0.0023**
eGFR baseline	78.68 (20.15)	78.03 (24.56)	78.77 (24.94)	103.41 (8.90)	75.73 (22.47)	79.15 (27.33)	78.80 (23.03)	**0.002**
Visual acuity (logMAR)	0.26 (0.20)	0.32 (0.22)	0.31 (0.22)	0.21 (0.17)	0.34 (0.19)	0.29 (0.23)	0.30 (0.21)	0.063
Spherical equivalent (D)	−0.55 (1.93)	−0.74 (1.93)	−0.79 (1.86)	0.75 (1.35)	−0.45 (1.09)	−1.30 (1.81)	−0.64 (1.85)	0.069
Total cholesterol (mmol/L)	4.29 (0.87)	4.37 (1.02)	4.24 (0.99)	4.55 (0.54)	4.24 (0.79)	4.11 (0.99)	4.30 (0.93)	0.058
Axial Length	23.95 (1.07)	23.96 (1.15)	24.02 (1.08)	22.89 (1.01)	23.76 (0.67)	23.71 (0.68)	23.92 (1.07)	0.056
Smoking status (yes, %)	52 (29.5)	54 (33.8)	29 (29.6)	6 (54.5)	10 (24.4)	4 (33.3)	155 (31.1)	0.61
SBP (mmHg)	142.81 (21.15)	144.03 (22.17)	140.69 (20.87)	145.77 (26.21)	145.59 (25.14)	155.23 (32.92)	143.38 (22.23)	0.48

*MAP* The Mean Arterial Pressure, *SBP* Systolic blood pressure, Glucose serum levels were measured during random testing.

The *p* values that are statistically significant (*p* < 0.05) are bolded.

**Fig. 1:**
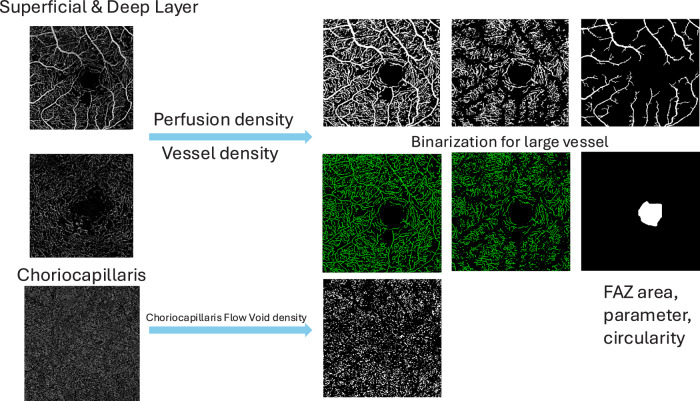
Representative pictures for OCTA analysing process.

We compared the OCTA metrics in both retina and choroid between DR severity groups. Table 2 shows OCTA quantitative metrics for retinal perfusion, FAZ and CC between groups. CC FD density, regardless of size, increased with DR severity with significant difference between groups, demonstrating higher FD density with increased DR severity (in all CC FD density: 17.06 vs 17.41 vs 17.60 vs 17.62 vs 18.05 vs 18.41, respectively, *p* trend=0.0004). In superficial FAZ, area and circularity’s trend showed difference between groups, PDR without PRP group has largest superficial FAZ area, longest perimeter and worst circularity perimeter. While severe group has the largest deep FAZ area between groups. In the superficial layer, the FAZ area was larger in the PDR w/o PRP group compared to the severe NPDR group (0.52 vs 0.40 mm^2^). Conversely, in the deep layer, the FAZ area was larger in the severe NPDR group than in the PDR w/o PRP group (2.28 vs 2.08 mm^2^). The results of the analysis comparing differences between individual groups are presented in Supplementary Table 1. Representative images of the OCTA data extraction process are shown in Fig. 1.

**Table 2:** Quantitative metrics for retinal perfusion, FAZ and choriocapillaris (CC).

OCTA metrics	No DR (*n* = 176)	Mild NPDR (*n* = 160)	Moderate NPDR (*n* = 98)	Severe NPDR (*n* = 11)	PDR with PRP (*n* = 41)	PDR without PRP (*n* = 12)	Total (*n* = 498)	p-trend
*CC flow voids density, %*								
All	17.06 (0.114)	17.41 (0.118)	17.60 (0.152)	17.62 (0.458)	18.05 (0.234)	18.41 (0.437)	17.41 (1.53)	**0.0004**
>200 μm^2^	15.38 (0.104)	15.78 (0.108)	16.01 (0.139)	16.24 (0.419)	16.42 (0.214)	16.88 (0.399)	15.77 (1.41)	**<0.0001**
>400 μm^2^	12.92 (0.116)	13.39 (0.12)	13.68 (0.154)	14.06 (0.464)	14.16 (0.237)	14.67 (0.443)	13.39 (1.58)	**<0.0001**
>600 μm^2^	10.99 (0.126)	11.51 (0.131)	11.82 (0.168)	12.23 (0.506)	12.4 (0.258)	12.99 (0.483)	11.51 (1.72)	**<0.0001**
>800 μm^2^	9.46 (0.132)	10.03 (0.137)	10.36 (0.176)	10.71 (0.53)	11.05 (0.271)	11.52 (0.505)	10.03 (1.81)	**<0.0001**
*Superficial FAZ*								
Area, mm^2^	0.32 (0.013)	0.39 (0.013)	0.41 (0.017)	0.40 (0.051)	0.41 (0.026)	0.52 (0.049)	0.38 (0.17)	**0.0027**
Perimeter, mm	1.34 (0.024)	1.44 (0.025)	1.46 (0.032)	1.33 (0.096)	1.49 (0.049)	1.66 (0.092)	3.03 (1.08)	0.739
Circularity	2.67 (0.079)	3.15 (0.082)	3.26 (0.105)	2.94 (0.317)	3.32 (0.162)	4.18 (0.303)	1.42 (0.32)	**0.0001**
*Deep FAZ*								
Area, mm^2^	1.48 (0.056)	1.71 (0.057)	1.8 (0.074)	2.28 (0.222)	1.85 (0.114)	2.08 (0.212)	1.68 (0.74)	**0.0022**
Perimeter, mm	1.64 (0.051)	1.62 (0.053)	1.55 (0.067)	1.23 (0.203)	1.43 (0.104)	1.44 (0.194)	7.02 (3.25)	**0.0012**
Circularity	6.91 (0.248)	7.28 (0.257)	7.02 (0.33)	6.4 (0.996)	6.66 (0.508)	6.88 (0.95)	1.59 (0.67)	0.6248
SCP_LV_PD, %	7.46 (0.111)	8.06 (0.115)	8.62 (0.147)	8.85 (0.443)	8.52 (0.226)	8.87 (0.423)	8.03 (1.52)	**<0.0001**
SCP_noLV_PD, %	19.15 (0.208)	18.4 (0.216)	17.17 (0.277)	17 (0.835)	16.84 (0.426)	19.15 (0.208)	18.08 (0.12)	**<0.0001**
SCP_PD, %	26.63 (0.15)	26.27 (0.155)	25.55 (0.199)	25.94 (0.6)	25.3 (0.306)	25.2 (0.572)	11.41 (2.64)	0.1646
SCP_LV_VD, %	3.6 (0.046)	3.8 (0.048)	4.02 (0.061)	4.34 (0.185)	4.12 (0.094)	4.24 (0.176)	3.82 (0.63)	0.2636
SCP_noLV_VD, %	15.89 (0.206)	15.08 (0.213)	13.93 (0.274)	13.14 (0.826)	13.41 (0.421)	12.95 (0.787)	14.81 (0.13)	0.0517
SCP_VD, %	19.63 (0.177)	18.98 (0.183)	18.06 (0.235)	17.6 (0.707)	17.66 (0.361)	17.36 (0.675)	18.85 (2.42)	**0.0239**
DCP _PD, %	9.52 (0.181)	8.54 (0.188)	7.83 (0.241)	8.47 (0.727)	7.96 (0.371)	8.34 (0.693)	8.69 (2.45)	0.0754
DCP_ VD, %	8.69 (0.172)	7.75 (0.179)	7.04 (0.229)	7.28 (0.691)	7.08 (0.353)	7.24 (0.659)	7.87 (2.34)	**0.0003**

All the means are adjusted by age.

*SCP* Superficial Capillary Plexus, *DCP* Deep Capillary Plexus, *VD* Vessel Density, *PD* Perfusion Density, *LV* large vessels, *no_LV* without large vessels, *DR* diabetic retinopathy, *FAZ* fovea avascular zone, *NPDR* non-proliferative diabetic retinopathy, *PDR* proliferative diabetic retinopathy.

The *p* values that are statistically significant (*p* < 0.05) are bolded.

We observed that with increased DR severity, large vessel perfusion density in the superficial layers showed significant differences between groups (7.46 vs 8.06 vs 8.62 vs 8.85 vs 8.52 vs 8.87, respectively, *p* trend<0.0001). The slope of the LV PD increased with DR severity, but flatten out once severe NPDR was reached. The overall retinal perfusion density trend showed no significant difference (*p* trend=0.16. In the entire retina, perfusion density decreased with DR severity (26.63 vs 26.27 vs 25.55 vs 25.94 vs 25.30 vs 25.20, respectively, p trend = 0.16), with the slope of decrease steeper in earlier stages or DR compare with the transition from moderate to severe NPRDR. (25.55–25.93%). Vessel density followed the same trend as perfusion density in the superficial layers, global density trend showed significant difference between groups (19.63 vs 18.98 vs 18.06 vs 17.6 vs 17.66 vs 17.36, respectively, *p* trend = 0.02).

**Fig. 2:**
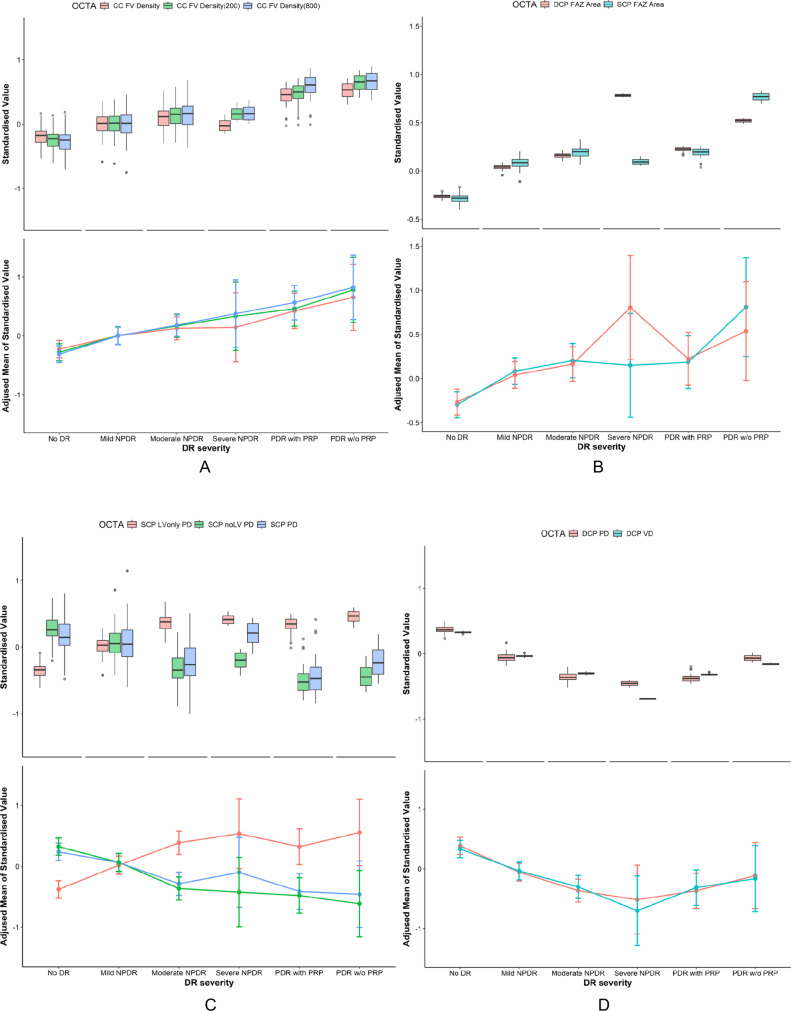
OCTA variables trend in DR verities. **A** CC FV density across groups. **B** FAZ area across groups. **C** Superficial layer perfusion and vessel density across groups. **D** Deep layer perfusion and vessel density across groups.

Trends of OCTA metrics in each group were showed in Fig. 2. CC FV increased with DR severity (Fig. 2A). The FAZ area in the deep layer was the largest in the severe NPDR group and whereas in the superficial layer it was largest in those with PDR. (Fig. 2B). The trend in the superficial layer showed that large vessels dilated and increased at the onset of the DR stage (Fig. 2C). PD and VD in deep layer showed similar change along the severity (Fig. 2D).

In Supplementary Table 2, we showed the correlation between BCVA, HbA1C and OCTA variables. We observed BCVA negatively correlated with CC FV density and superficial FAZ circularity (CC FV density Pearson’s ρ = 0.09, p = 0.04; superficial FAZ circularity Pearson’s ρ = 0.13, p = 0.003). Additionally, BCVA showed a negative correlation with both vessel density and perfusion density in the superficial and deep retinal layers. Meanwhile, we observed that HbA1C was not correlated with choriocapillaris variables or FAZ circularity in either the superficial or deep layers. However, HbA1C was negatively correlated with both perfusion density and vessel density in the superficial and deep layers. Axial length showed negatively correlated with PD superficial capillary plexus (superficial ρ = 0.09, *p* = 0.04). Meanwhile, sphere equivalent was negatively correlated with both PD and VD in deep layer (PD ρ = −0.1, p = 0.02; VD ρ = −0.1, p = 0.02).

In superficial layers, both global PD and PD without large vessel showed significant different between severe NPDR and laser group (p = 0.002, p = 0.006). Vessel density also showed difference between severe NPDR and laser group, in both global VD and VD without large vessels (p = 0.003, p = 0.01). Table 3

**Table 3:** Sub-group analysis between severe NPDR and PDR.

OCTA metrics	Severe NPDR N = 11	PRP done *N* = 41	No PRP N = 12	*P* value PRP vs. PDR without PRP	*P* value Severe NPDR vs. PRP	*P* value Severe NPDR vs. w/o PRP
*CC flow voids density, %*						
All	17.37 (1.53)	18.07 (1.45)	18.20 (1.65)	0.5217	0.1178	0.1335
>200 μm^2^	16.00 (1.65)	16.44 (1.58)	16.67 (1.59)	0.6611	0.4142	0.3287
>400 μm^2^	13.78 (1.83)	14.18 (1.96)	14.44 (1.81)	0.5636	0.3074	0.3927
>600 μm^2^	11.90 (1.93)	12.42 (2.16)	12.72 (2.01)	0.6071	0.3074	0.3293
>800 μm^2^	10.33 (1.98)	11.08 (2.24)	11.21 (2.14)	0.7932	0.2299	0.3205
*Superficial FAZ*						
Area, mm^2^	0.297	0.6899	0.2875	0.297	0.6899	0.2875
Perimeter, mm	2.88 (0.65)	3.33 (0.93)	4.13 (1.93)	0.0795	0.2137	0.0056
Circularity	1.31 (0.29)	1.49 (0.38)	1.64 (0.38)	0.1142	0.1602	0.0036
*Deep FAZ*						
Area, mm^2^	2.26 (0.62)	1.85 (0.69)	2.07 (0.87)	0.3672	0.0779	0.5452
Perimeter, mm	6.12 (1.99)	6.68 (2.78)	6.65 (1.75)	0.3599	0.583	0.2351
Circularity	1.17 (0.35)	1.44 (0.57)	1.39 (0.50)	0.7932	0.1348	0.5658
SCP_PD	13.35 (2.17)	11.05 (1.91)	11.98 (1.91)	0.1433	**0.0017**	**0.0268**
SCP_ LV_PD	8.66 (1.01)	8.53 (1.44)	8.72 (1.15)	0.9749	0.9121	0.8934
SCP_noLV_PD	7.83 (1.96)	6.06 (2.00)	6.73 (1.75)	0.1532	**0.0055**	0.1693
SCP_VD	11.10 (1.79)	9.41 (1.88)	10.06 (1.86)	0.2964	**0.0029**	0.2115
SCP_noLV_VD	7.51 (1.77)	6.13 (1.99)	6.64 (1.72)	0.2332	**0.0099**	0.2414
DCP_PD	8.78 (1.78)	7.93 (2.44)	8.59 (1.93)	0.398	0.2896	0.8088
DCP_VD	7.53 (1.50)	7.06 (2.23)	7.46 (1.64)	0.567	0.507	0.9075

*SCP* Superficial Capillary Plexus, *DCP* Deep Capillary Plexus, *VD* Vessel Density, *PD* Perfusion Density, *LV* large vessels, *no_LV* without large vessels.

The *p* values that are statistically significant (p < 0.05) are bolded.

## Discussion

We conducted a cross-sectional study to evaluate OCTA metrics across different DR severities and to analyse the correlations between OCTA metrics with visual acuity, HbA1C, DM duration, axial length and sphere equivalent. By using the non-invasive SS OCTA, we can quantify both the retina and choroid vascular changes in DR patients. Numerous studies have focused on FAZ and vascular changes in DR [9, 31]. However, vascular changes across all DR severities and correlation between OCTA capillary variables and patient’s systemic status remain unclear.

We found that increase CC flow voids density, FAZ area and decrease vascular perfusion was associated with more severe DR which was consistent with previous studies [32, 33]. Without dye leakage, OCTA is a more accurate and reproduceable method for quantifying the FAZ status [34]. We observed that a larger FAZ area and worse circularity were associated with increasing DR severity. Changes in the FAZ area in the deep layer occurred earlier than in the superficial layer, consistent with the changes observed in the CC. Meanwhile, trend of deep layer FAZ area showed significant steep trend in severe NPDR group, suggesting that deep layer FAZ area may change in earlier stage than superficial layer before progression to PDR.

The choriocapillaris exhibited worsening circulation with increasing DR severity, showing significant differences between DR groups. Our findings are consistent with previously published studies, which reported similar results regarding choroidal metrics and DR groups [23, 35]. Loss of choriocapillaris may compromise the blood supply to both the choroid and the outer retinal layers. In our PDR group, the choriocapillaris showed no significant difference between the laser-treated and non-laser-treated groups. This suggests that laser treatment in the peripheral regions may not cause significant change of the choriocapillaris in the macular region. We noticed that the trend in the choriocapillaris across increase severity of DR is similar to that of the FAZ parameters. Choriocapillaris dropout has been observed in diabetic eyes even in the absence of diabetic retinopathy (no-DR) and during the early stages of the disease [36]. Since the photoreceptors and RPE in FAZ primarily receives its blood supply from the choriocapillaris, circulatory changes in the choroid may be of greater significance compared to other regions [37]. CC flow void density has been identified as a biomarker for DR progression, can be correlated with DR severity, as demonstrated by Want et al. [38]. In a study by Viggiano et al. they found that in NPDR eyes, CC insufficiency were found to be associated with photoreceptor damage [39].

Our findings on early-stage DR OCTA changes are consistent with other research [34]. In the superficial retinal layer, overall perfusion density showed no significant differences between groups. However, the trend for VD and PD in large vessels increased from mild to moderate and severe NPDR, then decreased in the PDR group. This may indicate a significant response of large vessels prior in the early stages, with more ischemic changes occurring in the later stages of DR. In contrast, in the deep retinal layer, both PD and VD decrease rapidly in the early stages of NPDR (mild and moderate). This observation suggests that ischemic changes occur in the deep retinal layer early in the disease progression, while in the superficial layer, large vessels may exhibit ischemia in the later stages. Previous research has reported that perfusion in the deep retinal layer changes in the early stages and is more vulnerable to ischemic damage in DR, as the outer plexiform layer of the retina requires high oxygen levels [17, 40]. We have previously observed that loss of PD and VD in the peripheral retina in wide field OCTA occurs earlier in disease [13], these changes in the deeper capillary plexus may mirror the changes in the peripheral retina in early disease which should be further explored.

Both superficial and deep layer FAZ areas have been found to be negatively related to vision acuity. Additionally, loss of FAZ circulations and distortion of FAZ shape are associated with worse vision acuity in mild NPDR patients. This indicates that FAZ area, circulation and shape from OCTA screening may serve as good indicators of retinal function in DR patients [15, 41, 42]. In a 3-year longitudinal study, CC flow deficit is independently associated with DR progression [43]. Similarly, we observed that ischemia in the choriocapillaris blood flow was related to worse visual acuity in our study. This suggests that improving choroidal blood flow, or reducing the decline in diabetes, may be a viable therapeutic strategy for DR patients. We found both worse superficial and deep layer VD and PD are correlated with worse visual acuity, which was consistent with data reported by Hsiao et al. [44]. Disruption of the normal retinal capillary structure, reflected in the changes in VD and PD, may result in ischemia potentially affecting vision. Our results indicated that longer axial length, or higher sphere equivalent may lead to reduction of vessel density in that retina, which is consistent with prior studies [45, 46].

To further analyse the influence of PRP on OCTA in DR patients, we separated the PDR group into PDR with PRP and PDR w/o PRP subgroups. In PDR patients, PRP to ischemic peripheral retina can cause regression of NV and reduces progression of disease [47]. Russell and colleagues found small changes in capillaries and large vessels in a few eyes from their study after PRP treatment, but these changes were not statistically significant [48]. We similarly found no statistically significant differences between the group that had PRP compared to the fresh PDR without PRP.

Our research has several strengths and limitations. One key strength is that our analysis includes OCTA metrics not only in the retina but also in the choroid layer, providing a more comprehensive analysis of vessel changes in DR patients. These findings provide a better understanding of the disease pathology and may offer innovative ideas for early detection and treatment. We also analysed the correlations between patient characteristic and our OCTA metrics. Although these correlations were weak, our results still demonstrated some associations between visual acuity, HbA1C levels, and the OCTA metrics.

The severe NPDR group had a small sample size. Those with PRP present on recruitment were presumed to have had PDR. However, we conducted a subgroup analysis comparing the PRP group with those presenting with fresh PDR, since historically in our local clinical practice, physicians may elect to perform PRP early, in cases of severe NPDR where compliance with follow-up was a concern. Furthermore, this presumed PDR with PRP group had a much longer duration of diabetes compared with subjects presenting with active PDR which may result in differences in observe vascular metrics. While our study observed differences in choriocapillaris metrics across DR severities, it is essential to consider that normal topographic variations may also contribute to these measurements. Additionally, previous studies suggest that diabetic patients without clinical retinopathy can exhibit early CC changes, potentially impacting our findings [49, 50]. Further research comparing diabetic patients to healthy controls, as well as studies with larger scan areas, may help delineate pathological changes from normal variations.

Our study measured the FAZ area within the SCP and DCP, rather than across the whole retinal thickness. While whole-retinal FAZ measurements may provide a unified view and potentially reduce variability, SCP and DCP-specific measurements are still valuable [51, 52]. They allow for the identification of layer-specific ischemic changes, which may appear early in diabetic retinopathy and help us understand capillary-level alterations. Future studies should consider incorporating whole-retinal FAZ measurements to complement plexus-specific data and provide a more complete representation of FAZ alterations in DR. The observed differences in OCTA variable across DR severity groups, while statistically significant in some cases, are modest in magnitude. It is important to recognize that small effect sizes, particularly with marginal data, may have limited translate into clinically meaningful outcomes. These findings should be viewed as exploratory, emphasizing the need for future studies to validate their relevance in clinical practice and to determine their impact on patient management strategies.

Another limitation of our study is the lack of data on diabetic macular edema (DMO) status among participants. As a common complication of diabetic retinopathy, DMO can significantly influence OCTA measurements, may potentially impact the accuracy of our findings [53]. To mitigate this limitation, we have been cautious in our interpretation of OCTA-derived vascular metrics, acknowledging that part of the variability might stem from unmeasured edema effects. Future studies should ideally incorporate DMO assessment through OCT to control for its influence on vascular measurements, enabling more precise evaluation of ischemic and perfusion changes specific to DR severity.

The cross-sectional design of this study presents a limitation in interpreting the causality or progression of vascular changes across DR severities. As our data capture a single point in time for each patient, we are unable to determine whether the observed vascular changes precede or follow DR progression. Future longitudinal studies are necessary to track vascular alterations over time and to clarify the temporal relationship between these changes and DR progression. Such studies would allow for stronger causal inferences and a better understanding of the natural history of vascular alterations in diabetes. The OCTA images in our research were limited to a 3 × 3 mm² area, primarily focused on the central fovea, and therefore our findings on central retinal changes may not fully reflect changes occurring in the peripheral retina. In addition, the FAZ boundary in the deep layer is not as clear as in the superficial layer, which may introduce more variability in the measurements of the deep layer. Our study is a cross-sectional study, and a longitudinal study is required for a more comprehensive understanding of how OCTA vascular metric changes with DR progression.

## Conclusion

In conclusion, our research demonstrated that as DR severity increases, both retinal and choroidal vessels exhibit ischemic changes. According to OCTA metrics, the deep layer and large vessels in the superficial layer may change in the early stages of DR. Ischemic changes may be associated with worse visual acuity and higher HbA1C levels. PRP treatment in our cohort did not change central OCTA parameters in PDR patients.

## Summary

### What was known before:

- Prior research has demonstrated that OCTA can effectively quantify retinal and choroidal vascular changes in DR), particularly in terms of FAZ area enlargement, vascular perfusion decrease, and capillary dropout.

### What this study adds:

- Early Ischemic Changes in Deep Layer: The study highlights that ischemic changes in the deep retinal layer large vessel occur earlier and more rapidly compared to the superficial layer, suggesting the deep layer may be more vulnerable to early DR damage.
- Choriocapillaris and DR Severity: The findings confirm that worsening choriocapillaris circulation is strongly correlated with increasing DR severity, showing similar trends to FAZ parameters and suggesting the choriocapillaris plays a crucial role in DR progression.
- Visual Acuity Correlations: It shows a clear correlation between worse OCTA metrics (such as larger FAZ area, lower vessel density, and perfusion) and poorer visual acuity, making these OCTA parameters potential biomarkers for visual outcomes in DR patients.

## Supplementary information

Supplementary Table 1 Differences Between Individual Groups

Supplementary Table 2 OCTA metrics correlation with Studied variables

## Data Availability

The datasets used and/or analysed during the current study are available from the corresponding author on reasonable request.
