# Supplementary material for: Flow and ischemic changes in retina and choroid across diabetic retinopathy spectrum: a SS-OCTA study
Source: Eye (Lond). 2025 Feb 27;39(8):1631–40. doi: 10.1038/s41433-025-03639-y (PMC12089474; doi:10.1038/s41433-025-03639-y)
Supplement: Supplementary file 1 — Supplementary Table 1 Differences Between Individual Groups [file 41433_2025_3639_MOESM1_ESM.docx]

**Supplementary Table 1** Differences Between Individual Groups

| Variable | Mild_NPDR vs No DR | | Moderate_NPDR vs No_DR | | Severe_NPDR vs No_DR | | PRP vs No_DR | | No_PRP vs No_DR | |  |
| --- | --- | --- | --- | --- | --- | --- | --- | --- | --- | --- | --- |
|  | Effect Size(β) | P-value | Effect Size(β) | P-value | Effect Size(β) | P-value | Effect Size(β) | P-value | Effect Size(β) | P-value | |
| CC_FV_density | 0.2802 | 0.0901 | 0.4297 | **0.0245** | 0.252 | 0.5916 | 0.9524 | **0.0003** | 1.0765 | **0.0173** | |
| CC_FV_density_200 | 0.3374 | **0.0259** | 0.522 | **0.0029** | 0.5614 | 0.192 | 1.0081 | **<0.0001** | 1.2374 | **0.0028** | |
| CC_FV_density_800 | 0.4662 | **0.0157** | 0.729 | **0.0011** | 0.7787 | 0.1553 | 1.532 | **<0.0001** | 1.6563 | **0.0017** | |
| SCP_FAZ_area | 0.063 | **0.0007** | 0.0819 | **0.0001** | 0.0653 | 0.2123 | 0.0814 | **0.0054** | 0.1806 | **0.0004** | |
| DCP_FAZ_area | 0.2233 | **0.0051** | 0.3125 | **0.0007** | 0.7756 | **0.0006** | 0.3616 | **0.0043** | 0.5803 | **0.0077** | |
| SCP_LVonly_PD | 0.5568 | **0.0005** | 1.0812 | **<0.0001** | 1.1586 | **0.0107** | 1.0344 | **<0.0001** | 1.2201 | **0.0052** | |
| SCP_noLV_ PD | -0.5797 | 0.057 | -1.6967 | **<0.0001** | -1.3561 | 0.1173 | -2.2082 | **<0.0001** | -2.0122 | **0.0157** | |
| DCP _PD | -1.3523 | **0.0001** | -2.2996 | **<0.0001** | -2.6187 | **0.006** | -2.3609 | **<0.0001** | -1.3695 | 0.1337 | |
| DCP_VD | -1.2496 | **0.0007** | -2.1662 | **<0.0001** | -3.4999 | **0.0008** | -2.2199 | **0.0001** | -1.6604 | 0.0957 | |
|  |  |  |  |  |  |  |  |  |  |  | |

SCP: Superficial Capillary Plexus, DCP: Deep Capillary Plexus, VD: Vessel Density, PD: Perfusion Density, LV: large vessels, no_LV: without large vessels, DR, diabetic retinopathy; FAZ, fovea avascular zone; NPDR, non-proliferative diabetic retinopathy, PDR, proliferative diabetic retinopathy, The p-values that are statistically significant (p < 0.05) are bolded.
