# Supplementary material for: Flow and ischemic changes in retina and choroid across diabetic retinopathy spectrum: a SS-OCTA study
Source: Eye (Lond). 2025 Feb 27;39(8):1631–40. doi: 10.1038/s41433-025-03639-y (PMC12089474; doi:10.1038/s41433-025-03639-y)
Supplement: Supplementary file 2 — Supplementary Table 2 OCTA metrics correlation with Studied variables [file 41433_2025_3639_MOESM2_ESM.docx]

**Supplementary Table 2** OCTA metrics correlation with Studied variables

| Variables | CC flow voids density, % | Superficial FAZ area | Deep FAZ area | SCP PD | SCP VD | DCP PD | DCP VD |
| --- | --- | --- | --- | --- | --- | --- | --- |
| Visual acuity (logMAR) | 0.09* | 0.22* | 0.05 | -0.15* | -0.15* | -0.21* | -0.19* |
| HbA1C (%) | 0.07 | 0.02 | -0.06 | -0.1* | -0.1* | -0.14* | -0.13* |
| DM duration | -0.0018 | -0.07 | -0.01 | 0.001 | -0.03 | -0.002 | -0.02 |
| Axial Length | 0.07 | -0.15* | -0.06 | -0.09* | 0.02 | 0.02 | 0.05 |
| Sphere Equivalent | 0.001 | 0.08 | 0.07 | 0.06 | -0.04 | -0.1* | -0.14* |

*P<0.05

Adjusted for Age and DR severity
